# A Snack Dietary Pattern Increases the Risk of Hypercholesterolemia in Northern Chinese Adults: A Prospective Cohort Study

**DOI:** 10.1371/journal.pone.0134294

**Published:** 2015-08-05

**Authors:** Lixin Na, Tianshu Han, Wei Zhang, Xiaoyan Wu, Guanqiong Na, Shanshan Du, Ying Li, Changhao Sun

**Affiliations:** Department of Nutrition and Food Hygiene, Public Health College, Harbin Medical University, Harbin, China; The Ohio State University, UNITED STATES

## Abstract

The evidence about the effect of dietary patterns on blood cholesterol from cohort studies was very scarce. The study was to identify the association of dietary patterns with lipid profile, especially cholesterol, in a cohort in north China. Using a 1-year food frequency questionnaire, we assessed the dietary intake of 4515 adults from the Harbin People’s Health Study in 2008, aged 20-74 years. Principle component analysis was used to identify dietary patterns. The follow-up was completed in 2012. Fasting blood samples were collected for the determination of blood lipid concentrations. Logistic regression models were used to evaluate the association of dietary patterns with the incidence of hypercholesterolemia, hypertriglyceridemia, and low-HDL cholesterolemia. Five dietary patterns were identified (“staple food”, “vegetable, fruit and milk”, “potato, soybean and egg”, “snack”, and “meat”). The relative risk (RR) between the extreme tertiles of the snack dietary pattern scores was 1.72 (95% CI = 1.14, 2.59, *P* = 0.004) for hypercholesterolemia, 1.39 (1.13, 1.75, *P* = 0.036) for hypertriglyceridemia, after adjustment for age, sex, education, body mass index, smoking, alcohol consumption, energy intake, exercise and baseline lipid concentrations. There was a significant positive association between the snack dietary pattern scores and fasting serum total cholesterol (SRC (standardized regression coefficient) = 0.262, *P* = 0.025), LDL-c (SRC = 0.324, *P* = 0.002) and triglycerides (SRC = 0.253, *P* = 0.035), after adjustment for the multiple variables above. Moreover, the adjusted RR of hypertriglyceridemia between the extreme tertiles was 0.73 (0.56, 0.94, *P* = 0.025) for the vegetable, fruit and milk dietary pattern, and 1.86 (1.33, 2.41, *P* = 0.005) for the meat dietary pattern. The snack dietary pattern was a newly emerged dietary pattern in northern Chinese adults. It appears conceivable that the risk of hypercholesterolemia can be reduced by changing the snack dietary pattern.

## Introduction

Elevation of blood cholesterol concentrations has been recognized as a major risk factor for cardiovascular diseases [1]. Control of the increase in blood cholesterol is one of the important strategies for the prevention of cardiovascular diseases. Since the 1960s, recommendation to restrict dietary cholesterol has been adopted by many countries as dietary cholesterol was thought contributed to the blood cholesterol concentrations [2]. However, many epidemiological surveys in recent years have shown that there was no relationship between dietary cholesterol intake and coronary heart disease or blood cholesterol concentrations in various populations [3–5]. Therefore, the strategy of dietary prevention for hypercholesterolemia needs to be reconsidered.

In fact, blood cholesterol depends on many dietary factors, for example, dietary saturated fatty acids and fibers, at the same time [6]. These dietary factors collectively influence intestinal cholesterol absorption, hepatic cholesterol synthesis, and biliary excretion and cellular use. Investigation of single dietary cholesterol intake is inadequate for taking into account complicated interactions among all the nutrients or foods and their cumulative effects on blood cholesterol in studies of free-living people. In addition, people choose foods and combinations of foods rather than isolated nutrients, advice on nutrients intake has little practical effect for them. Dietary pattern analysis addresses the effect of diet as a whole and thus provides insight beyond the effects of single nutrient or food [7]. It can give the public more practical advice for the application of dietary prevention for hypercholesterolemia.

Some cross-sectional studies have addressed the association of dietary pattern with hypercholesterolemia or blood cholesterol. For example, a meat and fast-food pattern score was found associated with higher LDL-c in Korea [8]. In Dutch, a refined foods pattern was associated with higher total cholesterol concentration [9]. In Brazilian adults, a processed food pattern was positively associated with LDL-c, HDL-c, and total cholesterol values among the men [10]. Given the variety of dietary habits and cultures across the world, the identified patterns can be expected to differ by population, especially between the Eastern and the Western. The foreign dietary patterns have limited directions for the Chinese. Moreover, the cohort study about the association of dietary pattern with blood cholesterol is very limited. Therefore, it is necessary to clarify whether the daily dietary patterns influence the risk of hypercholesterolemia in the Chinese population with prospective cohort design.

The aim of this study was to identify and characterize dietary patterns from data collected using a 1-year food frequency questionnaire (FFQ) at baseline and clarify the prospective association of dietary patterns with lipid profile, especially cholesterol, in a cohort of adult residents in urban Harbin, north of China, by using principal component analysis (PCA). We identified 5 dietary patterns among the participants and found that a newly emerged dietary pattern, the snack dietary pattern, increased the risk of hypercholesterolemia during 4.2-year follow-up.

## Materials and Methods

### Participants and design

The participants were from the Harbin People’s Health Study (HPHS) who were recruited in 2008 [11]. HPHS was a population-based study focused on the topic of diet, nutrition and health. People were eligible to participate in HPHS if they were: 1) aged 20 y to 74 y, 2) without a history of using postmenopausal hormone therapy, malignancy, thyroid dysfunction, renal calculi, corticosteroid or calcitriol use. All the participants at baseline completed a detailed survey including an in-person interview for social status, living habits, chronic disease history, physical activity, dietary habit and measurement of anthropometric parameters. Current smokers were defined as those who have smoked at least 100 cigarettes lifetime and smoke every day or some days now. Current drinkers were defined as those who consumed ≥1 alcoholic drink each month in the 12 months before the survey. Regular exercise was defined as any kind of recreational or sport physical activity other than walking for work or life performed at least 30 minutes for three or more days per week. A total of 4,515 participants (50.5% of the whole study population) were randomly selected for the follow-up due to the limited financial resources. The first follow-up was completed in 2012, and the content of follow-up was as same as those at baseline. The fasting blood samples were collected at baseline and the follow-up for the measurement of blood lipid concentrations. Participants who had extreme values for total energy intake (< 500 or > 4500 kcal/d), or with more than 10 items unfilled in the questionnaire at baseline were excluded. In our analysis, we excluded participants with hypercholesterolemia at baseline. In addition, we also excluded participants with hypertriglyceridemia or low-HDL cholestterolemia at baseline, respectively, in order to analyze the association of dietary patterns with hypertriglyceridemia and low-HDL cholestterolemia during the follow-up.

### Ethics statement

The study was approved by the ethics committee of Harbin Medical University and has been conducted in accordance with the Declaration of Helsinki. Written informed consent was obtained from all participants.

### Dietary data collection

FFQ was administered to assess usual dietary intake in the past 12 months in our study. A total of 103 food items were included in the questionnaire, which covered most of the commonly consumed food in urban Harbin. For each food item, the subjects were asked how frequently they consumed over the preceding year, followed by a question on the amount consumed in lians (a unit of weight equal to 50 g) or ml (for liquid food item) per unit of time. The consumption frequency was transformed to obtain mean consumption a day. Nutrient intakes for each food item consumed were calculated by multiplying the nutrients content listed in the Chinese Food Composition Table [12]. The 103 food items in the FFQ were collapsed into 14 food groups, taking into account their nutritional characteristics (Table 1). Before the survey started, a random subsample of 147 healthy subjects were recruited to complete two FFQs (FFQ1 and FFQ2) and a 3-day dietary record (DR). The energy-adjusted correlation coefficient between the two FFQs was 0.60–0.77 for macronutrients, 0.53–0.75 for micronutrients, and 0.55–0.76 for major food groups. The energy-adjusted correlation coefficient between FFQ2 and DRs was 0.51–0.69 for macronutrients, 0.48–0.67 for micronutrients, and 0.45–0.66 for major food groups. When the above dietary nutrients and food groups were categorized by quartile, the proportion of subjects that were classified into the same quartile from the FFQ2 and DR was 38%-47%. Misclassification of subjects into the opposite quartile was 2%-6%. This indicates that the FFQ is a reliable and accurate method for assessing food and dietary nutrients intake.

**Table 1:** Food groups and food items from the FFQ in the study.

Food groups	Food items
Rice	Rice, foxtail millet and maize
Wheaten food	Noodle, steamed twisted roll and steamed bread, pancake, and bread
Potatoes and its products	Potato, sweet potato and vermicelli
Soybeans and its products	Tofu, dried bean curd and soybean milk
Vegetables	Mooli, garden radish, carrot, asparagus beans, soybean sprouts, sprouts of mung bean, eggplant, tomato, chili green, pimento, white gourd, cucumber, pumpkin, cocozelle, garlic bolt, garlic sprout, allium fistulosum, onions, Chinese chives, Chinese cabbage, sauerkraut, rape, flowering chinese cabbage, cabbage, red cabbage, cauliflower, broccoli, cabbage mustard, spinach, celery, leaf lettuce, coriander, crowndaisy chrysanthemum, baby Chinese cabbage, lettuce, mushroom, shii-take, black fungus, sea-tangle
Fruits	Apple, pear, peach, jujube, winter jujube, green grape, red grape, pomegranate, persimmon, strawberry, actinidia chinensis, orange, citrus, pomelo, pineapple, litchi, mango, banana, papaya, pitaya, durian, watermelon, Hami melon, melon, mangosteen
Livestock and its products	Pork, beef, mutton and offals (liver and intestine)
Poultry and its products	Chicken, duck, goose and offals
Milk	Cow's milk, yogurt and milk powder
Eggs	Egg
Seafood	Carp, crucian, hairtail, yellow croaker and shrimp
Snacks	Sugar-sweetened preserved fruits, biscuit, fried chips, chocolate, other sweets
Beverages	Sugar-sweetened drink, including fruit drink and fizzy drink
Icecream	Icecream

### Dietary pattern derivation

PCA was used to perform a dietary pattern analysis and to determine factor loadings based on the dietary exposure at baseline. Before analysis, all dietary variables were adjusted for energy intake using the residual approach [13]. Factors were rotated with varimax rotation to maintain uncorrelated factors and enhance interpretability. The number of factors to retain was chosen based on an eigenvalue > 1, scree plot test and factor interpretability. Five main factors were identified from the analysis. Factor loadings were calculated for each food group. Factors were thereby interpreted as dietary patterns and each pattern was named after the food group with the highest loading (absolute value of loading > 0.3). These loadings can be considered correlation coefficients between food groups and dietary patterns, and they take values between -1 and +1. We calculated for each participant a factor score for each of the 5 factors, the standardized intake of each food group was weighted by its factor loading.

### Anthropometric measurement and biochemical assessment

The height and weight were examined with participants wearing light, thin clothing and no shoes. Body mass index (BMI) was calculated as weight (kg) divided by the square of the height in meters (m^2^). Fasting blood samples were collected and concentrations of serum triglycerides, total cholesterol, LDL-c and HDL-c were measured using an automatic analyser (Hitachi, Japan).

### Study outcome definition

The study outcome was identified by self-report or by the following criteria [14]: Hypercholesterolemia: fasting total cholesterol ≥ 6.22 mmol/l, and/or fasting LDL-c ≥ 4.14 mmol/l; Hypertriglyceridemia: fasting triglycerides ≥ 2.26 mmol/l; low-HDL cholesterolemia: fasting HDL-c < 1.04 mmol/l (male) or fasting HDL-c < 1.29 mmol/l (female).

### Statistical analysis

Analysis were conducted in the year of 2013. For each dietary pattern identified, we categorized the factor scores into tertiles based on their distribution in the study population. The Logistic regression models were used to estimate the relative risk (RR) and 95% confidence interval (CI) for the middle and highest tertiles compared with the lowest tertile of each diet pattern, with adjustment for baseline values of age, sex, education, BMI, smoking, alcohol consumption, exercise and blood lipid concentrations. Multivariate linear regression analysis were performed using the dietary pattern score as independent variables and fasting lipid biochemistry parameters at follow-up as dependent variables. The standardized regression coefficients (SRCs) were adjusted for baseline values of age, sex, education, BMI, smoking, alcohol consumption, exercise and blood lipid concentrations. The difference among tertiles in demographic and lifestyle characteristics, was analyzed based on the χ^2^ test for categorical variables, and on the ANOVA for continuous variables. The difference among tertiles in nutrient intakes was analyzed by using ANCOVA with age, sex, BMI, smoking, alcohol consumption, exercise, and energy intake as covariants. The statistical analysis was performed using SPSS 21.0 (Beijing Stats Data Mining Co. Ltd, Beijing, China). Two sided *P* < 0.05 was considered statistically significant.

## Results

### Participants characteristics

**Fig 1:**
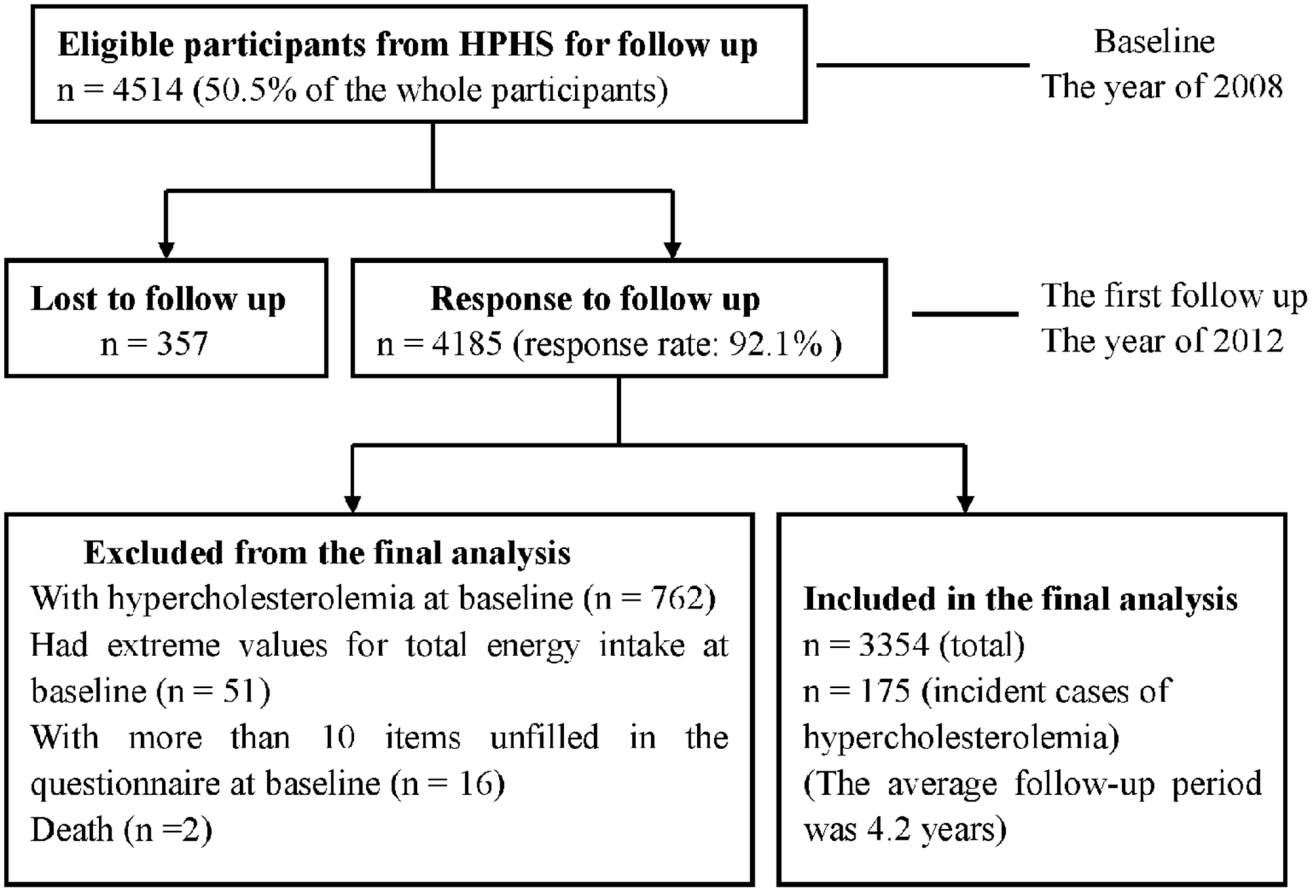
The flow chart of the participants.

Fig 1 shows the flow chart of the participants. A total of 4515 eligible participants were selected at baseline form HPHS for the follow-up in the year of 2008. And 4158 participants were followed with a response of 92.1% in 2012. Eight hundred and thirty-one participants were excluded from the analysis because they were with hypercholesterolemia, had extreme values for total energy intake, or with more than 10 items unfilled in the questionnaire at baseline. A total of 3354 participants were included in the final analysis in this study. The average follow-up period was 4.2 years. The number of the incident cases of hypercholesterolemia was 175. The demographic and biochemical characteristics of the incident cases of hypercholesterolemia and the control subjects at baseline were shown in Table 2. There were more women, current smokers in the cases of hypercholesterolemia and they were older, with lower education level, with higher concentrations of serum total cholesterol, LDL-c and triglycerides at baseline, compared with the control subjects.

**Table 2:** Description of demographic and biochemical characteristics of the incident cases of hypercholesterolemia and the control subjects at baseline. Data are means (SD) or n (%). Differences in categorical variables between the hypercholesterolemia and control groups in each study were analyzed by χ^2^ test. The mean levels of continuous variables between the 2 groups were tested by the independent-samples t test.

Characteristics	Hypercholesterolemia (n = 175)	Control (n = 3179)	*P* value
Male (%)	40 (22.9)	989 (31.1)	<0.001
Age (years)	54.2 (8.02)	50.2 (10.1)	0.014
BMI (kg/m^2^)	24.7 (3.43)	24.9 (3.40)	0.605
Education (%)			<0.001
No formal education	2 (1.14)	49 (1.54)	
Elementary school	4 (2.29)	172 (5.41)	
Middle school	66 (37.7)	1017 (32.0)	
High school/secondary technical school	56 (32.0)	1084 (34.1)	
Technical school/college	47 (26.9)	833 (26.2)	
Postgraduate degree or above	0 (0.00)	24 (0.75)	
Lifestyle factors (%)			
Current smoker	33 (18.9)	471 (14.8)	<0.001
Current drinker	52 (29.7)	937 (29.5)	0.391
Regular exercise	70 (40.0)	1539 (48.4)	<0.001
Blood lipids level			
Total cholesterol (mmol/l)	4.96 (1.02)	4.84 (0.95)	<0.001
LDL-c (mmol/l)	2.89 (1.05)	2.76 (0.92)	<0.001
HDL-c (mmol/l)	1.23 (0.35)	1.25 (0.31)	0.642
Triglycerides (mmol/l)	1.88 (1.67)	1.78 (1.42)	<0.001

Abbreviations: BMI, body mass index.

In analyzing the association of dietary patterns with hypertriglyceridemia, a total of 3047 participants were included at baseline. The number of the incident cases of hypertriglyceridemia was 189 after follow-up. In analyzing the association of dietary patterns with low-HDL cholesterolemia, a total of 2785 participants were included at baseline. The number of the incident cases of low-HDL cholesterolemia was 83 after follow-up. The demographic and biochemical characteristics of the incident cases and the control subjects at baseline were shown in S1 and S2 Tables, respectively.

### Dietary patterns

The scree plot of eigenvalues depicted 5 major dietary patterns for the participants in our study; the factor-loading matrices for those dietary patterns are list in Table 3. The larger the loading of a given food group to the factor, the greater the contribution of that food group to the specific factor. Negative loading indicated a negative association with the factor. The first dietary pattern, identified as staple food dietary pattern, was mainly loaded with food that made from rice and wheat. The second dietary pattern, identified as vegetable, fruit and milk dietary pattern, was heavily loaded with fresh vegetables, fruits, milk and its products. The third dietary pattern, identified as potato, soybean and egg dietary pattern, was loaded with potatoes, soybeans and eggs and their products. The fourth dietary pattern, identified as snack dietary pattern, was mainly loaded with biscuit, fried chips, liquid beverage, sweets and icecream. The fifth dietary pattern, identified as meat dietary pattern, was loaded with livestock and its organs, poultry and its organs, and seafood. These 5 patterns explained 51.3% of the variance in dietary pattern score.

**Table 3:** Factor loading for 5 food patterns for the participants in the study. Factor loading was obtained from the principal component analysis. Factor loadings ≥ 0.30 (30) are listed in the table.

Food groups	Rotated factor loading				
	Staple food pattern	Vegetable, fruit and milk pattern	Potato, soybean and egg pattern	Snack pattern	Meat pattern
Rice	0.79		-0.34		
Wheaten food	0.88				
Potatoes and its products			0.60		-0.37
Soybeans and its products			0.55		
Vegetables		0.56			
Fruits		0.70			
Livestock and its products					0.74
Poultry and its products					0.64
Milk		0.53			
Eggs			0.52		
Seafood		0.37			
Snacks		0.32		0.52	
Beverages				0.69	
Icecream			0.36	0.64	
% of explained variance	13.9	11.0	9.90	8.64	7.90
% of accumulated explained variance	13.9	24.9	34.8	43.5	51.3

### Association between dietary patterns and lipid profile

During the follow-up, higher snack dietary pattern scores predicted a higher risk of hypercholesterolemia (Table 4). When the highest and the lowest tertiles of the snack dietary pattern scores were compared, the RR of hypercholesterolemia was 1.72 (95% CI = 1.14, 2.59, *P* = 0.004), after adjustment for baseline values of age, sex, education, body mass index, smoking, alcohol consumption, energy intake, exercise and blood lipid concentrations. There was a significant positive association between the “snack” dietary pattern scores and fasting total cholesterol (SRC = 0.262, *P* = 0.025) and LDL-c (SRC = 0.324, *P* = 0.002) at follow-up, after adjustment for baseline values of age, sex, education, body mass index, smoking, alcohol consumption, energy intake, exercise and blood lipid concentrations (Table 5).

**Table 4:** RR (95% CI) of hypercholesterolemia on tertiles of energy-adjusted dietary pattern scores in the study. Model 1 was adjusted for age, sex; Model 2 was adjusted for the baseline values of age, sex, education, body mass index, smoking, alcohol consumption, energy intake, exercise and blood lipid concentrations.

	Tertiles of energy-adjusted dietary pattern score			
Variables	Low	Middle	High	*P* for trend
	RR(95% CI)	RR(95% CI)	RR(95% CI)	
Staple food pattern				
NO. of cases	61	59	55	
Model 1	1	0.98(0.69–1.39)	0.83(0.66–1.12)	0.39
Model 2	1	0.97(0.66–1.44)	0.76(0.62–1.05)	0.17
Vegetable, fruit and milk pattern				
NO. of cases	66	56	53	
Model 1	1	0.74(0.29–1.13)	0.65(0.43–1.08)	0.11
Model 2	1	0.70(0.38–1.12)	0.68(0.40–1.15)	0.21
Potato, soybean and egg pattern				
NO. of cases	52	50	55	
Model 1	1	0.98(0.69–1.40)	1.07(0.62–1.60)	0.35
Model 2	1	0.98(0.65–1.50)	1.01(0.66–1.53)	0.77
Snack pattern				
NO. of cases	44	58	71	
Model 1	1	1.24(0.98–1.72)	1.86(1.07–2.79)	0.001
Model 2	1	1.30(1.02–1.70)	1.72(1.14–2.59)	0.004
Meat pattern				
NO. of cases	48	55	54	
Model 1	1	1.29(0.86–1.94)	1.16(0.70–1.87)	0.62
Model 2	1	1.22(0.81–1.84)	1.08(0.70–1.65)	0.45

Abbreviations: CI, confidence interval; RR, relative risk.

**Table 5:** The association between the snack pattern scores and serum cholesterol levels at follow-up using multiple linear regression analysis.

	Total cholesterol		LDL-c		HDL-c	
	SRC	*P*	SRC	*P*	SRC	*P*
Snack pattern scores ^a^	0.283	0.017	0.355	<0.001	-0.023	0.301
Snack pattern scores ^b^	0.262	0.025	0.324	0.002	-0.025	0.268

Abbreviations: SRCs, standardized regression coefficients.

^a^ SRCs were adjusted for age and sex;

^b^ SRCs were adjusted for the baseline values of age, sex, education, body mass index, smoking, alcohol consumption, energy intake, exercise and blood lipid concentrations.

Moreover, the snack dietary pattern increased the risk of hypertriglyceridemia (S3 Table). The adjusted RR for the highest tertile, compared with the lowest tertile, was 1.39 (1.13, 1.75, *P* = 0.036). The vegetable, fruit and milk dietary pattern and the meat dietary pattern were also associated with the risk of hypertriglyceridemia (S3 Table). The adjusted RR for the highest tertile, compared with the lowest tertile, was 0.73 (0.56, 0.94, *P* = 0.025) and 1.86 (1.33, 2.41, *P* = 0.005), respectively. The snack dietary pattern scores (SRC = 0.253, *P* = 0.035) and the meat dietary pattern scores (SRC = 0.336, *P* <0.001) were positively associated with fasting triglycerides, while the vegetable, fruit and milk dietary pattern scores (SRC = -0.295, *P* = 0.014) were negatively associated with fasting triglycerides (S4 Table).There were no dietary patterns significantly associated with the risk of low-HDL cholesterolemia, although a trend of protective effect of the snack dietary pattern and the meat dietary pattern was observed (S5 Table). The staple food dietary pattern and the potato, soybean and egg dietary pattern were not associated with lipid profile.

### Demographic and lifestyle characteristics, and nutrient intakes according to energy-adjusted tertile scores of the snack dietary pattern

Participants with higher snack dietary pattern scores were more likely to be woman, younger, non-current smokers, non-current drinkers, with less regular exercise (all *P* < 0.05) (Table 6). And participants with higher snack dietary pattern scores took more energy, dietary fat and cholesterol, while took less dietary fiber, vitamin B1, folic acid and manganese, compared with those with lower snack dietary pattern scores (all *P* < 0.05).

**Table 6:** Baseline characteristics and nutrient intakes according to tertile scores of the snack dietary in the study. Data are means (SD) or n (%). Difference among tertiles were analyze by using ANOVA or χ2 test.

Variable	Tertiles in the snack pattern scores			*P* value
	Low	Middle	High	
Male (%)	427(38.2)	326(29.1)	276(24.7)	< 0.001
Age (years)	51.9(9.68)	51.5(9.24)	47.9(10.5)	< 0.001
BMI (kg/m^2^)	25.0(3.36)	24.9(3.48)	24.7(3.41)	0.058
Current smokers (%)	175(15.7)	170(15.2)	159(14.2)	0.029
Current drinkers (%)	363(32.5)	350(31.3)	276(24.7)	0.044
Regular exercise (%)	587(52.6)	537(47.9)	485(43.4)	0.011
Nutrient intakes				
Energy (Kcal/d) ^a^	2105(695)	2287(710)	2405(539)	< 0.001
Carbohydrates (g/d) ^b^	315(127)	344(135)	339(104)	0.337
Proteins (g/d) ^b^	65.5(31.5)	69.3(18.1)	70.8(31.4)	0.285
Fats (g/d) ^b^	67.0(21.0)	70.5(13.1)	86.1(22.4)	< 0.001
Cholesterol (mg/d) ^b^	312(225)	300(172)	393(311)	< 0.001
Fiber (g/d) ^b^	16.5(5.26)	12.3(5.43)	11.8(4.85)	< 0.001
Vitamin A (μg RE/d) ^b^	444(182)	437 (162)	464(207)	0.208
Vitamin E (mg/d) ^b^	10.4(4.12)	11.7(3.59)	11.5(5.13)	0.392
Vitamin C (mg/d) ^b^	107.4(56.9)	95.7(53.0)	105.3(63.5)	0.267
Vitamin B1 (mg/d) ^b^	17.0(8.34)	11.8(6.43)	10.6(6.67)	0.026
Vitamin B2 (mg/d) ^b^	0.95(0.35)	0.97(0.46)	0.92(0.34)	0.464
Folic acid (μg /d) ^b^	58.9(26.7)	44.6(22.0)	39.0(23.1)	< 0.001
Nicotinic acid (mg/d) ^b^	13.9(5.26)	13.8(4.84)	13.9(5.39)	0.315
Phosphorus (mg/d) ^b^	1205(444)	1101(308)	1082(473)	0.729
Calcium (mg/d) ^b^	510(222)	482(173)	503(269)	0.374
Magnesium (mg/d) ^b^	408(126)	307(95)	315(135)	< 0.001
Ferrum (mg/d) ^b^	21.8(8.11)	21.4(9.32)	25.7(6.48)	0.139
Cuprum (mg/d) ^b^	2.28(0.81)	2.27(1.01)	2.01(0.70)	0.405
Manganese (mg/d) ^b^	6.24(1.84)	4.42(1.38)	4.30(1.89)	< 0.001
Zinc (mg/d) ^b^	12.2(4.02)	10.2(4.64)	9.1(3.04)	0.294
Selenium (mg/d) ^b^	51.5(43.2)	50.2(31.2)	49.5(15.9)	0.682

Abbreviations: BMI, body mass index.

^a^ Difference among tertiles were analyze by using ANCOVA with age, sex, BMI, smoking, alcohol consumption and exercise as covariants.

^b^ Difference among tertiles were analyze by using ANCOVA with age, sex, BMI, smoking, alcohol consumption, exercise and energy intake as covariants.

## Discussion

The primary purpose of our study is to explore whether dietary patterns are associated with hypercholesterolema. We identified 5 dietary patterns using PCA that we described as “staple food dietary pattern”, “vegetable, fruit and milk dietary pattern”, “potato, soybean and egg dietary pattern”, “snack dietary pattern” and “meat dietary pattern”. The snack dietary pattern was newly emerged in China which was associated with a higher risk of hypercholesterolemia. In addition, the snack dietary pattern and the meat dietary pattern increased the risk of hypertriglyceridemia, while the vegetable, fruit and milk dietary pattern decreased the risk of hypertriglyceridemia.

Dietary pattern analysis has been widely applied to the studies focused on the effects of food and nutrition on health. The western dietary pattern, characterized by high consumption of high-fat food, processed meat, refined grains and sugar-based desserts; and the prudent dietary pattern, characterized by healthy food, including higher intake of vegetables and fruits, and lower intake of high-fat or fried food, were two mostly common dietary patterns identified associated with health [15–18]. In our study, the identified “snack dietary pattern” is not common as an independent pattern in the previous studies [19, 20]. It is a newly emerged dietary pattern in the northern Chinese population. It explained about 9% of the variation in dietary pattern score, reflecting a dietary pattern change in the Chinese.

The snack dietary pattern in our study included sugar-sweetened preserved fruits, biscuit, fried chips, chocolate, other sweets, sugar-sweetened drink, and icecream. It is similar with the “refined-foods” pattern in professor van’ study which was characterized by greater intakes of French fries, high-sugar beverages, and white bread and lesser intakes of whole-grain bread and boiled vegetables [9]. In that study, the “refined-foods” pattern was associated with higher total cholesterol concentrations [9], supporting our result observed in the follow-up that higher snack dietary pattern scores predicted a higher risk of hypercholesterolemia.

Studies have shown that consumption of snacks usually led to the higher intake of energy, fat, cholesterol and sodium [21, 22]. And in professor van’ study [9], the “refined-foods” pattern was associated with lower intakes of micronutrients. Therefore, we analyzed the nutrient intakes according to tertiles of the snack dietary pattern scores in order to provide some clues explaining possible mechanisms responsible for the association of snack dietary pattern with hypercholesterolemia. We found that participants with higher snack dietary pattern scores took more energy, dietary fat and cholesterol, while took less dietary fiber, vitamin B1, folic acid and manganese, compared to those with lower snack dietary pattern scores. Although the relationship between dietary cholesterol and blood cholesterol is still controversial, the dietary fat, especially saturated fatty acid, has been acknowledged to increase blood cholesterol concentrations [23]. In addition, dietary fiber, vitamin B1, folic acid and manganese have been recently found possibly play beneficial roles in blood cholesterol control. For example, besides the capacity of binding to bile acid, dietary fiber has been found to promote the formation of short-chain fatty acids, specifically propionate in its cholesterol-lowering effects in hypercholesterolemic adults [24, 25]. Vitamin B1 level has been found negatively correlated with lipid profile, and vitamin B1 supplementation effectively decreased serum total cholesterol concentrations in diabetic patients [26, 27]. Folic acid decreased cholesterol synthesis through reducing homocysteine level and low-dose folic acid supplementation has been found to lower concentrations of total cholesterol in subjects with atherosclerosis risk factors [28]. Dietary manganese intake was negatively correlated with serum total cholesterol in adults and manganese supplementation reduced the blood cholesterol levels in calcium-deficient ovariectomized rats [29, 30]. Based on the above studies, we hypothesize that the characteristics of these nutrients and the interaction among these nutrients in the snack dietary pattern mainly collectively contributed to the association of the snack dietary pattern with hypercholesterolemia.

In our study, the number of the dietary patterns identified was larger than most of previous studies [8, 10, 31]. This is probably because of the diversity of the Chinese food and cooking, as well as the large number of food items we included in the study. Besides the snack dietary pattern, the staple food dietary pattern and the potato, soybean and egg dietary pattern identified here were also not common in previous studies. These two dietary patterns were not associated with lipid profile. This probably because that most of the food items in these two dietary patterns contain less diet fat and cholesterol. For the egg in the potato, soybean and egg dietary pattern, it is a main source of dietary cholesterol. While many studies have found that egg consumption did not increase total cholesterol or LDL-c, and it might benefit to lipid profile in healthy individuals [32–35]. Among the 5 dietary patterns identified, the vegetable, fruit and milk dietary pattern and the meat dietary pattern were similar with the Prudent diet and the Western diet, respectively. But we did not observe the significant association of these two dietary patterns with blood cholesterol during the follow-up, which was different from most of the other studies [8–10]. One reason is probably that the large number of dietary patterns decreases the effect of each dietary pattern as the total effect of diet is certain. The other reason is possibly due to the relatively short follow-up period and the small number of incident cases, as we did find some trends between these two dietary patterns and the blood cholesterol.

We also analyzed the association of dietary patterns with blood triglycerides in our study. The association of the vegetable, fruit and milk dietary pattern and the meat dietary pattern with triglycerides were in agreement with other studies [9, 10]. This suggested that there is similarity to a certain degree in the association of dietary patterns with health between the Chinese and people living in other regions worldwide. The snack dietary pattern was also a risk pattern for hypertriglyceridemia. This is probably because that individuals with higher snack dietary pattern scores took more energy, dietary fat, while took less dietary fiber, vitamin and mineral, compared to those with lower snack dietary pattern scores [23, 26, 29, 36].

As a newly emerged dietary pattern, we analyzed the distribution of the snack dietary pattern in the population. Participants with higher snack dietary pattern scores were more likely to be woman, younger, non-current smokers, non-current drinkers, with less regular exercise. This indicated that the snack dietary pattern is more popular in women with younger age. And besides dietary factors, many other lifestyle factors, including smoking, alcohol consumption, and exercise may also influence the development of hypercholesterolemia. Therefore, it is necessary to promote the nutrition of snacks and the disadvantage of excessive intake of snacks. Comprehensive intervention including diet and other lifestyle factors should be an effective strategy for the prevention of hypercholesterolemia, and should be emphasized in the Chinese population.

Strengths of our study include the prospective study design, the rigorous methods of outcome ascertainment. There are also some limitations of our study. In the observational studies, the imperfections in dietary assessment have always been a concern. Although these imperfections are usually thought to be randomly distributed among categories of the outcome, systematic imperfections that could bias the risk estimates are a possibility. And the number of the incident cases was relatively small, which limited the competent subgroup analysis. In addition, our data were from the northern Chinese population. The dietary patterns can not been completely applied to southern China.

## Conclusions

In summary, we identified 5 main dietary patterns in the Chinese population, and found that a newly emerged dietary pattern, the snack dietary pattern, was associated with a higher risk of hypercholesterolemia and hypertriglyceridemia. The principle implication of our findings is that we validate the results from cross-sectional studies that dietary patterns influence blood cholesterol concentrations, and the risk of developing hypercholesterolemia may be reduced by changing the snack dietary pattern.

## Supporting Information

S1 Table Description of demographic and biochemical characteristics of the hypertriglyceridemia incident cases and the control subjects at baseline. (DOC)

S2 Table Description of demographic and biochemical characteristics of the low-HDL cholesterolemia incident cases and the control subjects at baseline. (DOC)

S3 Table RR (95% CI) of hypertriglyceridemia on tertiles of energy-adjusted dietary pattern scores in the study. (DOC)

S4 Table The association between the pattern scores and serum triglycerides at follow-up using multiple linear regression analysis. (DOC)

S5 Table RR (95% CI) of low-HDL cholesterolemia on tertiles of energy-adjusted dietary pattern scores in the study. (DOC)
