# Supplementary material for: A Snack Dietary Pattern Increases the Risk of Hypercholesterolemia in Northern Chinese Adults: A Prospective Cohort Study
Source: PLoS One. 2015 Aug 5;10(8):e0134294. doi: 10.1371/journal.pone.0134294 (PMC4526671; doi:10.1371/journal.pone.0134294)
Supplement: S1 Table — (DOC) [file pone.0134294.s001.doc]

**S1 Table. Description of demographic and biochemical characteristics of the h**[**ypertriglyceridemia**](http://dict.baidu.com/s?wd=hypertriglyceridemia)incident cases and the control subjects at baseline.

| **Characteristics** | **h**[**ypertriglyceridemia**](http://dict.baidu.com/s?wd=hypertriglyceridemia) **(n=189)** | **Control**  **(n=2858)** | ***P* value** |
| --- | --- | --- | --- |
| Male (%) | 85 (45.0) | 797 (27.9) | <0.001 |
| Age (years) | 53.5 (8.92) | 50.6 (9.35) | 0.024 |
| BMI (kg/m^2^) | 25.0 (3.15) | 24.7 (2.96) | 0.524 |
| Education (%) |  |  | <0.001 |
| No formal education | 3 (1.59) | 43 (1.50) |  |
| Elementary school | 9 (4.76) | 156 (5.46) |  |
| Middle school | 69 (36.5) | 909 (31.8) |  |
| High school/secondary technical school | 63 (33.3) | 952 (33.3) |  |
| Technical school/college | 43 (22.8) | 775 (27.1) |  |
| Postgraduate degree or above | 2 (1.06) | 23 (0.80) |  |
| Lifestyle factors (%) |  |  |  |
| Current smoker | 43 (22.8) | 383 (13.4) | <0.001 |
| Current drinker | 66 (34.9) | 817 (28.6) | <0.001 |
| Regular exercise | 80 (42.3) | 1340 (46.9) | 0.036 |
| Blood lipids level |  |  |  |
| Total cholesterol (mmol/l) | 4.94 (1.39) | 4.81 (1.50) | <0.001 |
| LDL-c (mmol/l) | 2.95 (0.94) | 2.80 (0.89) | <0.001 |
| HDL-c (mmol/l) | 1.17 (0.70) | 1.29 (0.54) | <0.001 |
| Triglycerides (mmol/l) | 1.90 (0.47) | 1.68 (0.36) | <0.001 |

Data are means (SD) or n (%). Differences in categorical variables between the hypercholesterolemia and control groups in each study were analyzed by χ^2^ test. The mean levels of continuous variables between the 2 groups were tested by the independent-samples t test.

Abbreviations: BMI, body mass index.
