# Supplementary material for: A Snack Dietary Pattern Increases the Risk of Hypercholesterolemia in Northern Chinese Adults: A Prospective Cohort Study
Source: PLoS One. 2015 Aug 5;10(8):e0134294. doi: 10.1371/journal.pone.0134294 (PMC4526671; doi:10.1371/journal.pone.0134294)
Supplement: S2 Table — (DOC) [file pone.0134294.s002.doc]

**S2 Table. Description of demographic and biochemical characteristics of the low-HDL cholesterolemia** incident cases and the control subjects at baseline.

| **Characteristics** | **low-HDL cholesterolemia (n=83)** | **Control**  **(n=2702)** | ***P* value** |
| --- | --- | --- | --- |
| Male (%) | 13 (15.7) | 1038 (38.4) | <0.001 |
| Age (years) | 49.7 (7.62) | 50.9 (11.4) | 0.218 |
| BMI (kg/m^2^) | 24.4 (3.25) | 24.9 (3.54) | 0.727 |
| Education (%) |  |  | <0.001 |
| No formal education | 1 (1.20) | 41 (1.52) |  |
| Elementary school | 5 (6.02) | 164 (6.07) |  |
| Middle school | 25 (30.1) | 875 (32.4) |  |
| High school/secondary technical school | 29 (34.9) | 902 (33.4) |  |
| Technical school/college | 23 (27.7) | 699 (25.9) |  |
| Postgraduate degree or above | 0 (0.00) | 21 (0.78) |  |
| Lifestyle factors (%) |  |  |  |
| Current smoker | 16 (19.3) | 408 (15.1) | <0.001 |
| Current drinker | 25 (30.1) | 760 (28.1) | 0.262 |
| Regular exercise | 35 (42.2) | 1324 (49.0) | <0.001 |
| Blood lipids level |  |  |  |
| Total cholesterol (mmol/l) | 4.80(0.97) | 4.86 (1.30) | 0.126 |
| LDL-c (mmol/l) | 2.74(0.82) | 2.85 (0.90) | <0.001 |
| HDL-c (mmol/l) | 1.08 (0.20) | 1.35 (0.57) | <0.001 |
| Triglycerides (mmol/l) | 1.86 (1.33) | 1.74(1.02) | <0.001 |

Data are means (SD) or n (%). Differences in categorical variables between the hypercholesterolemia and control groups in each study were analyzed by χ^2^ test. The mean levels of continuous variables between the 2 groups were tested by the independent-samples t test.

Abbreviations: BMI, body mass index.
