# Supplementary material for: A Snack Dietary Pattern Increases the Risk of Hypercholesterolemia in Northern Chinese Adults: A Prospective Cohort Study
Source: PLoS One. 2015 Aug 5;10(8):e0134294. doi: 10.1371/journal.pone.0134294 (PMC4526671; doi:10.1371/journal.pone.0134294)
Supplement: S3 Table — (DOC) [file pone.0134294.s003.doc]

**S3 Table. RR (95% CI) of h**[**ypertriglyceridemia**](http://dict.baidu.com/s?wd=hypertriglyceridemia) **on tertiles of energy-adjusted dietary pattern scores in the study.**

| **Variables** | **Tertiles of energy-adjusted dietary pattern score** | | | | | ***P* for trend** |
| --- | --- | --- | --- | --- | --- | --- |
|  | **Low** |  | **Middle** |  | **High** |  |
|  | **RR(95% CI)** |  | **RR(95% CI)** |  | **RR(95% CI)** |  |
| Staple food pattern |  |  |  |  |  |  |
| NO. of cases | 66 |  | 65 |  | 58 |  |
| Model 1 | 1 |  | 0.95(0.70-1.23) |  | 0.86(0.69-1.08) | 0.36 |
| Model 2 | 1 |  | 0.89(0.65-1.21) |  | 0.79(0.53-1.17) | 0.40 |
| Vegetable, fruit and milk pattern |  |  |  |  |  |  |
| NO. of cases | 75 |  | 66 |  | 48 |  |
| Model 1 | 1 |  | 0.85(0.72-1.02) |  | 0.65(0.40-0.93) | ＜ 0.001 |
| Model 2 | 1 |  | 0.77(0.60-0.98) |  | 0.73(0.56-0.94) | 0.025 |
| Potato, soybean and egg pattern |  |  |  |  |  |  |
| NO. of cases | 69 |  | 62 |  | 58 |  |
| Model 1 | 1 |  | 0.88(0.70-1.09) |  | 0.84(0.68-1.04) | 0.25 |
| Model 2 | 1 |  | 0.81(0.55-1.21) |  | 0.79(0.53-1.17) | 0.43 |
| Snack pattern |  |  |  |  |  |  |
| NO. of cases | 49 |  | 66 |  | 74 |  |
| Model 1 | 1 |  | 1.35(1.09-1.64) |  | 1.48(1.17-1.81) | 0.013 |
| Model 2 | 1 |  | 1.28(1.10-1.48) |  | 1.39(1.13-1.75) | 0.036 |
| Meat pattern |  |  |  |  |  |  |
| NO. of cases | 43 |  | 64 |  | 82 |  |
| Model 1 | 1 |  | 1.51(1.17-1.96) |  | 1.91(1.39-2.55) | ＜ 0.001 |
| Model 2 | 1 |  | 1.58(1.21-2.04) |  | 1.86(1.33-2.41) | 0.005 |

Model 1 was adjusted for age, sex; Model 2 was adjusted for the baseline values of age, sex, education, body mass index, smoking, alcohol consumption, energy intake, exercise and blood lipid concentrations.

Abbreviations: CI, confidence interval; RR, relative risk.
