# Supplementary material for: A Snack Dietary Pattern Increases the Risk of Hypercholesterolemia in Northern Chinese Adults: A Prospective Cohort Study
Source: PLoS One. 2015 Aug 5;10(8):e0134294. doi: 10.1371/journal.pone.0134294 (PMC4526671; doi:10.1371/journal.pone.0134294)
Supplement: S4 Table — (DOC) [file pone.0134294.s004.doc]

**S4 Table. The association between the pattern scores and serum triglycerides at follow-up using multiple linear regression analysis**.

|  | **Vegetable, fruit and milk pattern scores** | |  | **Snack pattern scores** | |  | **Meat pattern scores** | |
| --- | --- | --- | --- | --- | --- | --- | --- | --- |
|  | **SRC** | ***P*** |  | **SRC** | ***P*** |  | **SRC** | ***P*** |
| Triglycerides ^a^ | -0.361 | <0.001 |  | 0.274 | 0.022 |  | 0.350 | <0.001 |
| Triglycerides ^b^ | -0.295 | 0.014 |  | 0.253 | 0.035 |  | 0.336 | <0.001 |

^a^ SRCs were adjusted for age and sex; ^b^ SRCs were adjusted for the baseline values of age, sex, education, body mass index, smoking, alcohol consumption, energy intake, exercise and blood lipid concentrations.

Abbreviations: SRCs, standardized regression coefficients.
