# Supplementary material for: A Snack Dietary Pattern Increases the Risk of Hypercholesterolemia in Northern Chinese Adults: A Prospective Cohort Study
Source: PLoS One. 2015 Aug 5;10(8):e0134294. doi: 10.1371/journal.pone.0134294 (PMC4526671; doi:10.1371/journal.pone.0134294)
Supplement: S5 Table — (DOC) [file pone.0134294.s005.doc]

**S5 Table. RR (95% CI) of low-HDL cholesterolemia on tertiles of energy-adjusted dietary pattern scores in the study.**

| **Variables** | **Tertiles of energy-adjusted dietary pattern score** | | | | | ***P* for trend** |
| --- | --- | --- | --- | --- | --- | --- |
|  | **Low** |  | **Middle** |  | **High** |  |
|  | **RR(95% CI)** |  | **RR(95% CI)** |  | **RR(95% CI)** |  |
| Staple food pattern |  |  |  |  |  |  |
| NO. of cases | 26 |  | 27 |  | 30 |  |
| Model 1 | 1 |  | 1.02(0.69-1.50) |  | 1.17(0.76-1.81) | 0.74 |
| Model 2 | 1 |  | 1.05(0.70-1.49) |  | 1.18(0.73-1.93) | 0.65 |
| Vegetable, fruit and milk pattern |  |  |  |  |  |  |
| NO. of cases | 23 |  | 28 |  | 32 |  |
| Model 1 | 1 |  | 1.33(0.80-2.22) |  | 1.46(0.94-2.28) | 0.24 |
| Model 2 | 1 |  | 1.21(0.70-2.13) |  | 1.37(0.85-2.19) | 0.59 |
| Potato, soybean and egg pattern |  |  |  |  |  |  |
| NO. of cases | 30 |  | 27 |  | 26 |  |
| Model 1 | 1 |  | 0.94(0.72-1.16) |  | 0.92(0.71-1.15) | 0.38 |
| Model 2 | 1 |  | 0.91(0.70-1.13) |  | 0.90(0.69-1.14) | 0.43 |
| Snack pattern |  |  |  |  |  |  |
| NO. of cases | 29 |  | 28 |  | 26 |  |
| Model 1 | 1 |  | 0.97(0.86-1.09) |  | 0.91(0.80-1.03) | 0.25 |
| Model 2 | 1 |  | 0.98(0.87-1.10) |  | 0.95(0.85-1.06) | 0.31 |
| Meat pattern |  |  |  |  |  |  |
| NO. of cases | 31 |  | 27 |  | 25 |  |
| Model 1 | 1 |  | 0.91(0.87-0.98) |  | 0.89(0.79-1.01) | 0.11 |
| Model 2 | 1 |  | 0.95(0.89-1.03) |  | 0.92(0.83-1.06) | 0.27 |

Model 1 was adjusted for age, sex; Model 2 was adjusted for the baseline values of age, sex, education, body mass index, smoking, alcohol consumption, energy intake, exercise and blood lipid concentrations.

Abbreviations: CI, confidence interval; RR, relative risk.
